# Rationale, design and conduct of a comprehensive evaluation of a school-based peer-led anti-smoking intervention in the UK: the ASSIST cluster randomised trial [ISRCTN55572965]

**DOI:** 10.1186/1471-2458-5-43

**Published:** 2005-04-22

**Authors:** Fenella Starkey, Laurence Moore, Rona Campbell, Mark Sidaway, Michael Bloor, ASSIST (A Stop Smoking In Schools Trial)

**Affiliations:** 1 Department of Social Medicine, University of Bristol, Bristol, England, UK; 2 Cardiff Institute of Society, Health and Ethics, Cardiff University, Cardiff, Wales, UK; 3 Faculty of Social Sciences, University of Glasgow, Glasgow, Scotland, UK

## Abstract

**Background:**

To date, no school-based intervention has been proven to be effective in preventing adolescent smoking, despite continuing concern about smoking levels amongst young people in the United Kingdom. Although formal teacher-led smoking prevention interventions are considered unlikely to be effective, peer-led approaches to reducing smoking have been proposed as potentially valuable.

**Methods/design:**

ASSIST (A Stop Smoking in Schools Trial) is a comprehensive, large-scale evaluation to rigorously test whether peer supporters in Year 8 (age 11–12) can be recruited and trained to effect a reduction in smoking uptake among their fellow students. The evaluation is employing a cluster randomised controlled trial (RCT) design with secondary school as the unit of randomisation, and is being undertaken in 59 schools in South East Wales and the West of England. Embedded within the trial are an economic evaluation of the intervention costs, a process evaluation to provide detailed information on how the intervention was delivered and received, and an analysis of social networks to consider whether such a peer group intervention could work amongst schoolchildren in this age group.

Schools were randomised to either continue with normal smoking education (n = 29 schools, 5562 students), or to do so and additionally receive the ASSIST intervention (n = 30 schools, 5481 students). No schools withdrew once the trial had started, and the intervention was successfully implemented in all 30 schools, with excellent participation rates from the peer supporters. The primary outcome is regular (weekly) smoking, validated by salivary cotinine, and this outcome has been obtained for 94.4%, 91.0% and 95.6% of eligible students at baseline, immediate post-intervention, and one-year follow-up respectively.

**Discussion:**

Comprehensive evaluations of complex public health interventions of this scale and nature are rare in the United Kingdom. This paper demonstrates the feasibility of conducting cluster RCTs of complex public health interventions in schools, and how the rigour of such designs can be maximised both by thorough implementation of the protocol and by broadening the scope of questions addressed in the trial by including additional evaluative components.

## Background

### Smoking and young people in the United Kingdom

Smoking rates amongst 11 to 16 year-olds in the United Kingdom continue to be a cause for concern, with increases in these during the early to mid-1990s [1,2]. Since the late 1990s, rates have stabilised at around 10 per cent [3-6]. Nonetheless, there is a very strong policy initiative from the British Government to lower these rates to 9 per cent or less by the year 2010 [7]. The means by which such a downward trend could be achieved, however, are unclear as to date no intervention has been shown, via rigorous evaluation, to be effective in reducing smoking uptake or encouraging smoking cessation amongst young people [8-10].

### Peer-led interventions

Many anti-smoking programmes for young people are school-based, due to the 'captive audience' at which such interventions can be targeted over several years [11-13]. However, evidence suggests that formal teacher-led interventions are unlikely to be effective in reducing smoking [14,15]. In contrast, peer-led approaches, particularly those which take an informal educational approach, have been identified as potentially promising for use in anti-smoking initiatives [16,17].

### ASSIST (A Stop Smoking in Schools Trial)

This paper reports upon the design of ASSIST (A Stop Smoking in Schools Trial), a large-scale comprehensive evaluation of an intervention which used such an informal peer-led approach to smoking prevention in schools. The intervention approach used by ASSIST was developed in a feasibility study undertaken in Wales [18] and was based upon the 'diffusion of innovation' model [19]. This has as its exemplar the US 'gay hero' sexual health promotion programme [20], whereby the diffusion of new behavioural norms through social networks was effected by locally influential opinion-formers. The promising results of the feasibility study led the Medical Research Council (MRC) to fund a full-scale evaluation of the intervention. This paper describes and discusses the design of this evaluation and key issues in its successful implementation.

## Methods/design

### Study design

**Figure 1:**
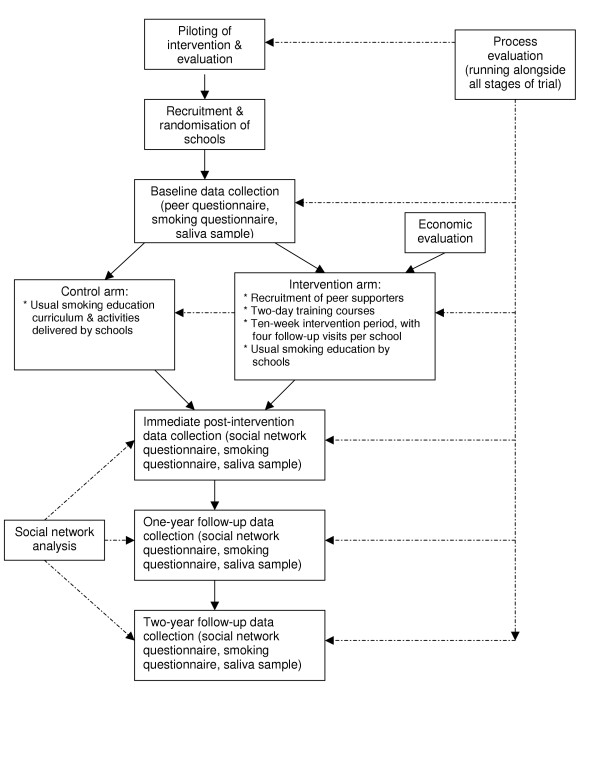
A Stop Smoking in Schools Trial (ASSIST) research design

While a randomised controlled design is being used to test the intervention's effectiveness, it was recognised that this would not allow full and in-depth exploration of the complex processes operating in such an informal peer-led intervention. The incorporation of several other components within the randomised controlled trial, including a detailed process evaluation, an economic evaluation, and an analysis of social networks, has resulted in a comprehensive evaluation that is enabling the study team to answer questions beyond those of effectiveness (see Figure 1).

Since the focus of the intervention was peer groups within a school year group, the unit of randomisation for evaluation of the intervention must be the school and a cluster randomised design has therefore been adopted.

### The ASSIST intervention

The ASSIST intervention trained influential Year 8 students (age 11–12) to use informal contacts with peers in their school year group to encourage them not to smoke. All students in participating schools were asked to nominate students they viewed as influential in different respects, and those nominated students were invited to train to take on the role of 'peer supporter'. Training for these peer supporters was undertaken by health promotion trainers (who had themselves been trained to deliver the intervention in a standardised way). The two-day training course for peer supporters was provided for each school at a venue away from the school premises, and aimed to: increase knowledge about the health, economic, social and environmental risks of smoking; emphasise the benefits of remaining smoke-free; and encourage the development of skills to enable peer supporters to promote non-smoking among their peers. After this training, peer supporters were asked to intervene informally in everyday situations over a ten-week period to encourage their peers not to smoke, and were asked to keep a diary record of these informal conversations. Four follow-up visits to each school were made by the health promotion trainers over this ten-week period to provide further training to the peer supporters and to monitor their progress. Students who submitted a completed diary at the end of this ten-week period were given a £10 gift voucher in recognition of their contribution to the project. This intervention is described in more detail in Audrey *et al *[21].

The intervention design, formative evaluation and feasibility study for ASSIST were undertaken over a two-year period [18]. Further refinement of the intervention, and of data collection procedures and instruments, was undertaken by the ASSIST team during additional pre-trial piloting work in three schools outside the final trial's geographical areas. This additional piloting work was required to refine the peer supporter nomination process in order to recruit more male peer supporters, to update elements of the intervention, and to undertake a 'dress rehearsal' of both the intervention and evaluation with the new teams involved.

### Selection, recruitment and randomisation of schools

In order to achieve representative and generalisable results, the design of ASSIST embraces as wide a range of secondary school settings as possible, including Welsh medium schools, private (fee-paying) schools (attended by a substantial fraction of students in the West of England area), religious schools, single-sex schools, and both urban and rural schools (serving populations with varying levels of social deprivation).

Letters and information sheets explaining the study's aims were sent to head teachers of 223 secondary schools in the former counties of Avon (England), Gwent, Mid Glamorgan, South Glamorgan and West Glamorgan (Wales). Schools expressing an interest (n = 127) were then visited individually by study team members. These meetings were conducted with a member of the school's senior management team, and included detail on the commitment required from schools. The importance of the randomised design was emphasised, and it was made clear to schools that it would be preferable for schools to decline participation than to join the study and then withdraw at a later point. Six schools withdrew before random selection and a further eight were excluded from the trial (since they had fewer than 60 students in Year 8, were special schools, or were involved in another anti-smoking initiative).

One hundred and thirteen schools remained interested in participating, from which the 66 schools required were randomly selected. The 113 schools were divided into four groups: (1) eight independent schools; (2) four Welsh medium schools; (3) four single-sex schools; and (4) 97 mixed-sex state schools. From each of the first three groups, 50 per cent of schools were randomly selected, giving eight schools. The 97 state schools were stratified by country (Wales or England) and whether the proportion of students entitled to free school meals was above or below the national averages (as an indicator of the socio-economic status of the school catchment area). Within these strata, schools were then listed according to Year 8 size. A systematic random sample was taken using a one-in-two sampling fraction, giving a further 50 schools. Two state schools were then randomly selected from each of the four strata defined by country and free school meal entitlement, to bring the total sampled to 66.

After this selection and before randomisation into trial arms, schools were sent a detailed memorandum of understanding to sign, to obtain head teachers' written commitment to the trial. Fifty-nine of these schools returned this document and thereby agreed to be randomised to either the intervention or control arm of the trial.

Stratified randomisation was then used to allocate these schools to either intervention or control group. Within each stratum, each school had a 50 per cent chance of being allocated to either group. Strata were defined using similar criteria to the random selection procedure described above, namely (1) independent schools, (2) Welsh medium schools, and (3) state schools. The two single-sex schools were not treated as a separate stratum since one was a boys' school and the other a girls' school. State schools were further grouped into eight strata, defined by (i) whether they were in Wales or England, (ii) whether they had a year size greater or less than the median (200 students), and (iii) whether they had greater or less than the median proportion of students entitled to free school meals (19%). To ensure concealment, the sequence in which schools were allocated was determined by one of the trial's principal investigators. Unaware of which school was the next to be allocated, a second principal investigator used a pocket calculator to generate a random number which determined the next school's group allocation.

### Outcome measures and sample size calculations

The trial's primary outcome measure is smoking prevalence among the high-risk group, i.e. those who, at baseline, had experimented with cigarettes, were ex-smokers, or were occasional (less than weekly) smokers. These students have been chosen as the primary target group because they are at greatest risk of becoming regular smokers, and the feasibility study showed an effect amongst this group [18]. Smoking prevalence is defined as students smoking a cigarette in the previous seven days. A secondary outcome measure is smoking prevalence among the entire year group. These outcome measures are being validated by measurement of salivary cotinine (a metabolite of nicotine), as studies have found cotinine to be the most accurate biomarker of smoke exposure in the previous two to three days [22,23]. Other secondary measures include perceptions of norms regarding adolescent smoking, and intention to quit.

There were 59 schools at baseline data collection, with a mean year size of 187 students. On average, 38.6 per cent (n = 72) of the year group were in the high-risk group of students who, at baseline, had experimented with cigarettes, or were ex-smokers, or were occasional (less than weekly) smokers. The intra-cluster correlation of weekly smoking at baseline, and also of the proportion in the high-risk group, was 0.025. Based on these data, the trial has 80 per cent power to detect a 5.8 per cent difference in weekly smoking among the high-risk group, assuming that weekly smoking prevalence among the high-risk group in control schools at 12-month follow-up is 23 per cent. Among all students, the trial has 80 per cent power to detect a 4.3 per cent difference in weekly smoking, assuming control group prevalence at 12-month follow-up is 15 per cent.

### Data collection

In order to obtain these outcome data, smoking behaviour was assessed at baseline with further assessments immediately post-intervention, and then at one year and two years post-intervention. At baseline, all students were asked to complete a questionnaire on smoking and a peer questionnaire, and to provide a saliva sample both for cotinine assay and to minimise reporting bias [24]. The smoking questionnaire drew upon questions about smoking used in the large-scale Office for National Statistics surveys of young people in England [25] and in the World Health Organisation's European Health Behaviour of School-Aged Children Study [26], to enable the results of ASSIST to be compared to national and international findings.

The peer questionnaire asked students to supply names of Year 8 students whom they viewed as influential in different ways. The results were used to identify students to invite to be peer supporters in intervention schools (although the purpose of this activity was not disclosed to students at the time of completion to avoid bias), and to identify a group of potential peer supporters in control schools for comparative purposes.

Subsequent data collection sweeps involve students completing a smoking questionnaire and a social network questionnaire (described later in this paper), and providing a saliva sample. The same core questions on smoking status are used at each follow-up to provide comparable outcome data, while additional questions are included at different follow-ups to explore secondary research questions.

All staff undertaking data collection have been provided with a data collection protocol and have been given training to maximise standardisation of data collection procedures across the trial sites and data collection sweeps.

### Statistical analyses

Data analysis is being conducted following a pre-specified analysis plan, agreed by the trial's independent Data Monitoring and Ethics Committee. Confidence intervals for smoking prevalence among all students and among the high-risk group are being estimated using design-weighted survey estimators implemented in Stata, which account for the clustering of students in schools. Estimates of the intervention effect are being obtained at student level using random effects logistic regression models with school as random effect, and all models include the five school-level stratifying variables as covariates (these five variables being binary variables indicating [i] independent school, [ii] less than 19 per cent of students entitled to free school meals, [iii] in England, [iv] Welsh medium, and [v] fewer than 200 students in Year 8).

As well as the student-level analyses reported above, the analysis plan includes school-level analyses that treat the data as repeated cross-sections rather than as an individual student-level cohort. Although they may have less statistical power, these analyses are less likely to suffer from non-response bias, and mean that analysis and inference takes place at the same level as the unit of randomisation and implementation. School-level analysis is being undertaken using appropriately weighted multiple regression analysis of the logarithm of each school's smoking prevalence at baseline and follow-up. Multi-level modelling is also being used to identify any important interactions between school-level factors and student-level effects.

In addition to the design and implementation of this large-scale cluster randomised trial to rigorously test the intervention's effectiveness, further components are nested within the evaluation to enable a comprehensive assessment of the intervention's impact in schools.

### Additional components of evaluation

#### Process evaluation

A detailed process evaluation is being undertaken by dedicated staff which aims to: examine the implementation and receipt of the intervention and its evaluation; explore the context and intensity of the intervention; and document factors external to the intervention which might impact upon both its implementation and its effectiveness. Both qualitative and quantitative methods are being used to collect data from key participants (students, teachers, health promotion trainers and researchers) at each stage of the intervention and evaluation. More details of the process evaluation design and implementation can be found in Parry-Langdon *et al *[27].

#### Economic evaluation

An economic evaluation is also being undertaken alongside the trial, relating costs to a range of outcomes in the form of a costs and consequences analysis. Although such analysis is not evaluative in the sense of informing technical or allocative efficiency, it is the most common form of economic study used in health care [28].

Costing is being undertaken using standard methods [29]. As the aim is to estimate the cost of replicating the intervention elsewhere, all costs associated with the evaluation are being excluded. Resources used have been recorded on a weekly basis by ASSIST staff using standardised forms, and include staff time, travel time and distance, consumables, accommodation, and vouchers for peer supporters.

#### Analysis of social networks

As the ASSIST intervention relies upon diffusing new behavioural norms through existing social networks within the school year group, an important component of the evaluation is to collect data on the nature of participating students' social networks. These data, obtained via questionnaires completed at the post-intervention data collection sweeps, are being used to explore the structural properties of teenage friendship groups (using social network analysis software). The social network data allow examination of the degree to which peer supporters were proximal in social space to those in the high-risk group. In combination with data from the immediate post-intervention follow-up, they also permit analysis of whether students' awareness of the intervention's existence was associated with their having a friendship link with a peer supporter and whether being a peer supporter had any impact on their friendship groups.

Consideration of variability in social networks amongst young people (for example the extent to which young people spend time with those in the same year group, or in other year groups) enhances our understanding of potential differences in the intervention's success in participating schools, therefore contributing to a more comprehensive picture of whether and under what conditions such peer-led diffusion-based interventions might succeed in affecting young people's behaviour.

### Participation rates

#### Participation of schools

ASSIST attracted a high level of interest among the secondary schools initially contacted. No school that participated in baseline data collection withdrew from the project other than due to enforced closure (n = 2). The training and four follow-up sessions comprising the intervention were also successfully implemented in all intervention schools (n = 30).

#### Participation of students

**Figure 2:**
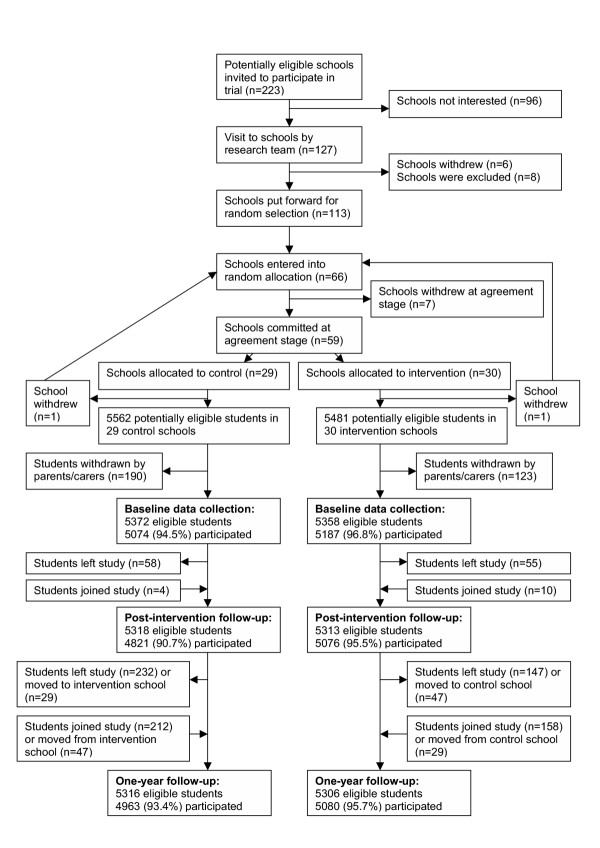
Flow of students through trial up to one-year follow-up data collection

Response rates for data collection sweeps are very high, as outlined in Figure 2.

The slightly lower participation rate at immediate post-intervention follow-up was due to the postal method of absentee data collection used only at this sweep, which yielded lower returns.

With regard to the students invited to be peer supporters (n = 942), 92 per cent (n = 867) of those invited agreed to attend the training course. Eight hundred and forty eight young people attended this training and 835 (98%) of them then consented to undertake the intervention as peer supporters (with an equal gender balance of 418 boys and 417 girls). Eighty-two per cent (n = 687) of the peer supporters completed and submitted their diary as requested, suggesting that such an intervention can be successfully implemented in a school setting.

As can be seen, extremely high levels of participation are being maintained throughout the study, demonstrating that comprehensive evaluations of this kind can be successfully conducted in secondary school settings if adequate rigour is adopted and appropriate support provided to both schools and participating students.

## Discussion

### Key issues in conduct of evaluation

Particular issues arise in conducting such an evaluation in secondary school settings, which are likely to be of interest to researchers considering undertaking similar work. These issues include how to minimise contamination across trial arms, how to develop and maintain positive working relationships with schools, how to obtain consent from participants, and how to undertake data collection in schools sensitively and successfully.

#### Contamination

In order to minimise contamination of control schools and their students with information and awareness of the intervention, and to prevent declining commitment to the project among control schools, all schools are being asked not to publicise their involvement in the trial until after the one-year follow-up data collection. Neither schools nor local health promotion agencies have been informed which schools are participating in the trial. In this way, the study team aims to ensure that details of the intervention, for example training activities undertaken, are not provided to control schools and used by teachers as part of their health education activities.

#### Involving schools in a randomised controlled trial

Peterson *et al *[9] suggest key principles for developing and maintaining positive collaborative research relationships with schools, which include keeping schools informed, minimising the work burden upon schools, maintaining regular contact with schools, executing tasks as planned, demonstrating sensitivity and responsiveness to schools' needs, and expressing appreciation (p.154). These are all principles used by the ASSIST teams to maximise the recruitment and retention of schools.

For example, after randomisation each participating school received a planning visit from the research team, conducted with the head or deputy head teacher and a designated school contact. These meetings involved discussion of: information provision to school governors, staff and parents; planning for study activities; responsibilities of the school contact person; process evaluation activities; provision to schools of payment for teacher cover and administration; and media issues and confidentiality of study participants. This meeting was intended to emphasise the school's commitment to participating in a rigorously conducted trial, but also to focus upon ways in which the study teams aimed to place a minimal burden on schools involved. Schools' individual needs and circumstances have been taken into account as far as possible (for example if extra data collection staff would be required to provide support in schools with high numbers of students with learning difficulties).

This planning stage started the process of building good working relationships with school staff who are acting as key contacts for the study teams throughout the trial. Relationships with these key school staff are being maintained via regular contact, newsletters to schools on the study's progress, and the execution of all study activities as planned and agreed with schools (to avoid disruption to their timetabling processes).

#### Consent

Obtaining consent for participation in such a community-based trial with young people entails a different process to that which might be used in a drug trial with adult participants. The first phase of consent procedures implemented in ASSIST was to ask head teachers to give written consent for the school's participation in the trial, prior to randomisation. Subsequently (and at least two weeks before baseline data collection), standardised information letters were posted by schools (not distributed via students) to parents/carers of all Year 8 students, explaining the study and enclosing a reply slip to return if they did not want their child to participate. Parents/carers were also given the opportunity to contact the research team at any time to discuss the trial, and a total of fifteen did so.

This passive, 'opt-out' method of parental permission has been found to be an ethical and appropriate way of informing parents/carers of such 'low-risk' prevention research, and to avoid the problems of low response rate, significant sampling bias and under-reporting of illicit activities encountered in research which has used active consent procedures with parents/carers of young people involved in school-based research [9,30].

In order to ensure acceptability of such passive parental permission procedures, these should be combined both with an opportunity for young people to refuse to participate in research, and with detailed procedures to safeguard confidentiality of data [31]. The third phase of consent procedures therefore occurs at the data collection visits, when all students are given an information leaflet (the contents of which are explained to them) and provided with the opportunity to ask questions about the study. They are then asked to complete and sign an assent form that gives them the option to either take part in or refuse some or all of the activities. Confidentiality safeguards at data collection sweeps are described below. These procedures are administered individually for all new students at subsequent data collection sweeps and have been agreed with the Wales Multi-Centre Research Ethics Committee (MREC), which reviewed the trial protocol.

#### Undertaking data collection in schools

It has proved crucial to adopt 'user-friendly' data collection methods with participating students in order to build trust and assure confidentiality, thereby maximising participation and accuracy of the data collected.

As recommended by educational researchers as good practice [9,31], questionnaires and saliva samples are collected in schools by study staff, either in classrooms with individual groups or in halls with larger numbers of students. Teachers are asked to be present but not to become involved in the data collection process itself, to reassure students that their answers are not seen by school staff. At the start of each data collection session, study staff provide an assurance of confidentiality to students, explaining that their individual results are only seen by university staff and are not given either to the school or to their parents/carers. An assurance of anonymity is also provided, with an explanation that both their questionnaires and salivette are marked with an identification number so that their names are not required on these items, and that only university staff are able to link these identification numbers with participating students' names.

The questionnaire completion procedures are explained at the start of the session, and the saliva collection procedure is explained and demonstrated by study staff prior to the administration of this activity. Students are encouraged to ask questions throughout the session if they do not understand or are unclear about any questionnaire items. Teachers are asked to identify students with reading or concentration difficulties who might require extra support from study staff to participate. When students have completed their questionnaires, they seal these into individual envelopes and return them directly to study staff, to reinforce their confidential nature.

To maximise participation, study staff return to each school approximately two weeks after the data collection session to collect data from students who were absent. The data collection methods described above are also used at these absentee sessions. An alternative method of collecting data from absent students was tested during the immediate post-intervention data collection sweep, whereby school staff were given absent students' questionnaires and asked to arrange their completion and return (by 'freepost'). This method was found to be less effective in maximising participation rates and its use has therefore been discontinued.

Such carefully designed data collection procedures, which aim to maximise young people's confidence in the confidential and anonymous nature of the data provided, should be regarded as good practice when undertaking school-based research. As can be seen from the participation rates outlined above, these procedures are leading to very high response rates from students.

Evaluation results will be presented in future publications as they become available. However, it is hoped that the clear exposition of the study design and methodology in this paper, reinforced as good practice by the high recruitment and participation rates being achieved, will help to inform planning and implementation of future school-based cluster randomised trials, and facilitate the design of comprehensive, rigorous evaluations of complex public health interventions.

## Competing interests

The author(s) declare that they have no competing interests.

## Authors' contributions

FS: drafted the paper, participant in the design of research tools, data collection, and analysis of the data.

LM: participant in the design, co-ordination, data collection and statistical analyses, and involved in revising the paper for important intellectual content.

RC: participant in the design, co-ordination, data collection and statistical analyses, and involved in revising the paper for important intellectual content.

MS: participant in the design of research tools and data collection, contributed to a draft of this paper.

MB: participant in the design, co-ordination and data collection, substantial contributor to the development of the experimental intervention, and involved in revising the paper for important intellectual content.

All authors read and approved the final manuscript.

## Members of the ASSIST team

*Lead Investigators*: Laurence Moore (CISHE, Cardiff University), Rona Campbell (Department of Social Medicine, University of Bristol), Nina Parry-Langdon (Health Promotion Division, Welsh Assembly Government), Mick Bloor (Faculty of Social Sciences, University of Glasgow).

*Bristol evaluation team*: Fenella Starkey, Suzanne Audrey

*Cardiff evaluation team*: Mark Sidaway, Jo Holliday.

*Health promotion trainers*: Kathleen Cordall, Lin Cooper, Heather Anderson-Paine, Nicola Hewer, Lorna Coombes, Rob Sage.

## Pre-publication history

The pre-publication history for this paper can be accessed here:


