# Rationale, design and conduct of a comprehensive evaluation of a school-based peer-led anti-smoking intervention in the UK: the ASSIST cluster randomised trial [ISRCTN55572965]

**DOI:** 10.1186/1471-2458-7-301

**Published:** 2007-10-23

**Authors:** Fenella Starkey, Laurence Moore, Rona Campbell, Mark Sidaway, Michael Bloor, ASSIST (A Stop Smoking In Schools Trial)

**Affiliations:** 1 Department of Social Medicine, University of Bristol, Bristol, UK; 2 Cardiff Institute of Society, Health and Ethics, Cardiff University, Cardiff, UK; 3 Faculty of Social Sciences, University of Glasgow, Glasgow, UK

In preparing the main results paper from this trial, we have noticed an important error in the study protocol paper[1]. In the paragraph headed 'Outcome measures and sample size calculations', the current text "A secondary outcome measure is smoking prevalence among the entire year group." is incorrect, since smoking prevalence among the entire year group is one of two primary outcomes, rather than a secondary outcome. The equal status of the two primary outcomes was agreed at a meeting of the Trial Steering Committee on the 21^st ^January 2005, where the analysis plan was finalised prior to completion of trial data collection. As indicated in the sample size paragraph, study power was considered in terms of both the whole year group and the high-risk sub-group. The incorrect emphasis in the published study protocol paper on the high risk sub-group analysis was a legacy of the funding proposal protocol, in which the sub-group sample size calculations critically determined the number of schools required for the trial.

Some other changes to the paragraph are also required to contextualise the corrected status of the outcomes. The corrected version of the paragraph should read:

## Outcome measures and sample size calculations

The trial's primary outcome measure is smoking prevalence measured (i) among the high-risk group and (ii) among the entire year group. The high-risk group is defined as those students who, at baseline, had experimented with cigarettes, were ex-smokers, or were occasional (less than weekly) smokers. These students are a primary target group because they are at greatest risk of becoming regular smokers, and the feasibility study showed an effect amongst this group [18]. Smoking prevalence is defined as students smoking a cigarette in the previous seven days. These outcome measures are being validated by measurement of salivary cotinine (a metabolite of nicotine), as studies have found cotinine to be the most accurate biomarker of smoke exposure in the previous two to three days [22,23]. Secondary outcome measures include perceptions of norms regarding adolescent smoking, and intention to quit.

We apologise for any inconvenience caused in our oversight with regard to this important detail.

## Pre-publication history

The pre-publication history for this paper can be accessed here:


